# 2020, A weird year!

**DOI:** 10.1002/jsp2.1136

**Published:** 2020-12-28

**Authors:** Mauro Alini, Rob Mauck, Daisuke Sakai

**Affiliations:** ^1^ AO Research Institute Davos Switzerland; ^2^ McKay Orthopaedic Research Laboratory University of Pennsylvania Philadelphia Pennsylvania USA; ^3^ Department of Orthopaedic Surgery Tokai University School of Medicine Isehara Kanagawa Japan

This year was a weird one, for science and especially for our lives! It will take some time to get back, or better, to enter a new “normality.” While this transition is happening in the different corners of our planet, at different speeds, we are pleased to present you with our last issue of 2020!

In spite of this bizarre year, we have received more than 75 submissions (reaching our target!), making this the highest number of submissions across the 3 years that the Journal has been in existence. This indicates that the *JOR Spine* is becoming the journal to submit in spine‐related research.

We are also extremely happy to announce the second *JOR Spine* Early Career Award, with the support of the ORS Spine Section. The award recognizes excellent young investigators in our field and highlights their outstanding work published in *JOR Spine*. This year looks also to be a particular one, as we had two indivisible winners. We are happy to announce the recipients of this year's award to be Dr Zehra Uruj and Dr Zhen Li for their manuscripts “Spinopelvic alignment predicts disc calcification, displacement, and Modic changes: Evidence of an evolutionary etiology for clinically‐relevant spinal phenotypes” and “ Proinflammatory intervertebral disc cell and organ culture models induced by tumor necrosis factor alpha,” published in the March and September 2020 issues, respectively. We are looking for a same tight competition for the next year's award.

Also, we are excited to highlight this special issue featuring the collected papers from the ORS PSRS Fifth International Spine Research Symposium, “New Horizons in Intervertebral Disc Research,” which took place the 2019 fall at Skytop Lodge in Pennsylvania. This issue features cutting edge reviews and primary research from invited speakers and awardees at the meeting, with a special editorial and introduction by Chitra Dahia, James Iatridis, and Lachlan Smith.

Finally, in our effort to involve and to reach out different countries, we are also pleased to announce to our Chinese colleagues and friends, the launch of another “Regional Reviews” series, focusing this time on spine researchers from China, with Guest Editors, Prof. Zhuojing Luo, Prof. Bin Li and Assoc. Prof. Zhen Li.

As the *JOR Spine* expands and increases its readership and leadership, we are also entering a critical phase in which our initial impact factor will be issued by mid‐2022. This is an important and a unique turning point for the journal and for the whole spine research community as well. Therefore, we would like to thank, in advance, all our submitters and followers for their future nonstop support.

With our best seasonal greetings and enjoy the issue!

